# KNOW-Ped CKD (KoreaN cohort study for outcomes in patients with pediatric CKD): Design and methods

**DOI:** 10.1186/s12882-016-0248-0

**Published:** 2016-03-25

**Authors:** Hee Gyung Kang, Hyun Jin Choi, Kyung Hee Han, Seong Heon Kim, Hee Yeon Cho, Min Hyun Cho, Jae Il Shin, Joo Hoon Lee, Joongyub Lee, Kook Hwan Oh, Young Seo Park, Hae Il Cheong, Curie Ahn, Il-Soo Ha

**Affiliations:** Department of Pediatrics, Seoul National University Children’s Hospital, Seoul, South Korea; Department of Pediatrics, Jeju University Hospital, Jeju, South Korea; Department of Pediatrics, Pusan National University Children’s Hospital, Yangsan, South Korea; Department of Pediatrics, Samsung Medical Center, Sungkyunkwan University School of Medicine, Seoul, South Korea; Department of Pediatrics, Kyungpook National University School of Medicine, Daegu, South Korea; Department of Pediatrics, Yonsei University College of Medicine, Severance Children’s Hospital, Seoul, South Korea; Department of Pediatrics, Asan Medical Center, University of Ulsan College of Medicine, Seoul, South Korea; Department of Preventive Medicine, Seoul National University Hospital, Seoul, South Korea; Department of Internal Medicine, Seoul National University Children’s Hospital, Seoul, South Korea

**Keywords:** Chronic kidney disease, Cohort study, Design, Prognostic factor, Asian children

## Abstract

**Background:**

The global prevalence of chronic kidney disease (CKD) is increasing. In children, CKD exhibits unique etiologies and can have serious impacts on children’s growth and development. Therefore, an aggressive approach to preventing the progression of CKD and its complications is imperative. To improve the understanding and management of Asian pediatric patients with CKD, we designed and launched KNOW-Ped CKD (KoreaN cohort study for Outcome in patients With Pediatric Chronic Kidney Disease), a nationwide, prospective, and observational cohort study of pediatric CKD with funding from the Korean government.

**Methods/design:**

From seven major centers, 450 patients <20 years of age with CKD stages I to V are recruited for the comprehensive assessment of clinical findings, structured follow-up, and bio-specimen collection. The primary endpoints include CKD progression, defined as a decline of estimated glomerular filtration rate by 50 %, and a requirement for renal replacement therapy or death. The secondary outcomes include the development of left ventricular hypertrophy or hypertension, impairment of growth, neuropsychological status, behavioral status, kidney growth, and quality of life.

**Discussion:**

With this study, we expect to obtain more information on pediatric CKD, which can be translated to better management for the patients.

**Trial registration:**

NCT02165878 (ClinicalTrials.gov), submitted on June 11, 2014.

## Background

Globally and locally, the prevalence of chronic kidney disease (CKD) is increasing along with prolonged lifespans and over-nutrition in many areas of the world [1]. The development of genomics and health technology has enabled genetic diagnoses of rare and common diseases and interventions for improving the outcome of previously untreatable diseases, such as congenital nephrotic syndrome of the Finnish type. Additionally, the life expectancy of cancer survivors and extremely low birth weight preterm infants has continued to improve, not infrequently along with secondary CKD. Therefore, the numbers of children with CKD of both primary and secondary causes have increased, and the proportion of CKD in renal patients is increasing.

To treat failed kidneys, nephrologists use powerful tools, namely dialysis and allograft kidney transplantation, to keep patients alive. However, the long-term prognosis and quality of life of patients who depend on renal replacement therapy (RRT) are poor, and healthcare expenses related to RRT are rapidly increasing along with the number of patients with end-stage renal disease (ESRD) [2]. Therefore, numerous studies have aimed to slow or stop the progression of CKD, including observational, prospective cohort studies of large numbers of participants to establish evidence for intervention strategies [3–6]. Given that the majority of these studies have been conducted in adult CKD patients, it is uncertain whether the results of the studies can be applied to pediatric patients [7]. First, the etiology of pediatric CKD is different from that of adult patients. Whereas diabetic nephropathy and hypertensive nephropathy are major causes of CKD in adults, congenital anomalies of kidney and urinary tract (CAKUT) are the most common causes of CKD in children. Children with CKD often suffer from impaired growth and development in addition to common complications of CKD, such as cardiovascular and metabolic bone disease [8–15]. Furthermore, the life expectancy of pediatric CKD patients is much longer than that of adult CKD patients; therefore, pediatric patients require a more aggressive approach to slowing the progression of CKD and reducing its complications [16–20]. Fortunately, excellent studies have investigated pediatric CKD, and many issues concerning pediatric CKD are being resolved [16, 21–24]. However, the majority of these studies are from Western countries with small proportions of Asian individuals.

We introduce a pediatric cohort study in Korea, KNOW-Ped CKD (KoreaN cohort study for Outcome in patients With Pediatric Chronic Kidney Disease), a nationwide, ten-year prospective, observational cohort study of pediatric CKD that was launched in 2011 as a sub-cohort of KNOW-CKD, which was funded by the Korean government [6]. The aim of the study is to answer the following key questions and optimize the care of patients with pediatric CKD: What are the common etiologies of pediatric CKD in Korea? What factors affect disease progression? What impact does pediatric CKD have on patients’ and their families’ lives? Is the current management for pediatric CKD ideal? Do medications, socio-economic status, or other modifiable factors influence the course of pediatric CKD?

## Methods/design

Seven major pediatric nephrology centers in Korea have participated in the study and enroll 450 children with CKD for comprehensive assessments of clinical findings, structured follow-up, and bio-specimen collection. To obtain follow-up data from more than five years, the patients are recruited during the first five years of this study. This study was approved by the institutional review boards of the participating centers, namely Seoul National University Hospital, Asan Medical Center, Kyungpook National University Hospital, Severance Hospital, Samsung Medical Center, Jeju University Hospital, and Pusan National University Children’s Hospital, and is included with the designation NCT02165878 at clinicaltrials.gov (submitted on June 11, 2014). The annual reports summarizing the data of this study will be available at our website (www.knowkidney.org) upon completion of data collection.

### Study population

Children <20 years of age with CKD are enrolled in this study after informed consent is obtained from parents or guardians. CKD is defined and staged according to the Kidney Disease Improving Global Outcomes (KDIGO) guidelines as either kidney damage or glomerular filtration rate (GFR) <60 mL/min/1.73 m^2^ for ≥3 months (Table 1) [25, 26]. GFR was estimated using the bedside CKiD formula [27]. For those <2 years old, operational staging of CKD was defined by the authors considering age-appropriate normal values of estimated GFR (eGFR), as shown in Table 1 [25, 28, 29].

**Table 1:** CKD staging according to age (eGFR, mL/min/1.73 m^2^)

Stage/Age	<2 weeks	2–7 weeks (<2 months)	2–23 months (<2 years)	≥2 years
1	≥30	≥49	≥72	≥90
2	20–29	33–48	48–71	60–89
3	10–19	16–32	24–47	30–59
4	5–9	8–15	12–23	15–29
5	<5	<8	<12	<15

The inclusion and exclusion criteria are presented in Table 2. CKD stage I with potentially progressive renal diseases or bilateral renal anomalies/defects is included, and CKD stage V, which requires maintenance renal replacement therapy, is excluded.

**Table 2:** Inclusion and exclusion criteria

Inclusion	Exclusion
Age, 0 to 19 years	Unable or unwilling to provide consent
CKD stages I to V according to KDIGO guidelines	Renal replacement therapy
Informed consent	Pregnant women
	Current cancer

### Data collection

After obtaining informed consent, the following clinical information is collected: medical history, anthropometric measurements, laboratory tests and imaging studies according to the schedule presented in Table 3. The etiology of CKD is determined from clinical findings, classified according to the North American Pediatric Renal Trials and Collaborative Studies (NAPRTCS) database [21] after modification, and categorized into one of the following three groups: CAKUT, glomerulonephropathy, and other. For the analysis of CKD progression rate, two time points are recorded along with eGFR on those dates; the date on which the diagnosis of etiologic kidney disease was made and the date when deterioration of renal function (CKD stage 3) was observed. The questionnaires of the Korea National Health and Nutrition Examination Survey are completed by the participants and their parents at the time of baseline assessment as part of the medical history [30]. The parents’ heights and the age of maternal menarche are recorded to assess the growth of the participants. Current medications included prescribed medications, over-the-counter medications taken for more than one month, and alternative medications.

**Table 3:** Study measurements by visit

	Parameter	Screening	Baseline	1 y	2 y	3 y	4 y	5 y	6 y	7 y	8 y	9 y	10 y
General	Informed consent	O											
	Demographic information	O											
	Medical history	O											
	KNHANES questionnaire		O										
	Recent events			O	O	O	O	O	O	O	O	O	O
	Medications		O	O	O	O	O	O	O	O	O	O	O
Kidney	CBC, blood/urine chemistry		O	O	O	O	O	O	O	O	O	O	O
	Kidney ultrasonography^a^		O	O	O	O	O	O	O	O	O	O	O
	Isotopic GFR (optional)		O			O			O			O	
Growth	Height, weight, waist/hip ratio, Tanner stage		O	O	O	O	O	O	O	O	O	O	O
	Bone age		O	O	O	O	O	O	O	O	O	O	O
Cardio-vascular	Blood pressure		O	O	O	O	O	O	O	O	O	O	O
	Electrocardiogram, chest X-ray		O		O		O		O		O		O
	Echocardiography		O					O					O
	24-hour ambulatory blood pressure monitoring (optional)		O	O		O		O		O		O	
Metabolic bone disease	Parathyroid hormone, vitamin D		O	O		O		O		O		O	
	Bone mineral density		O					O					O
Neuro-cognitive	Pediatric quality of life		O			O			O			O	O
	IQ test^b^			O			O			O			O
	Behavioral assessment^c^				O			O			O		
	Parental bonding instrument^d^		O										O
Bio-repository	Genetic sample		O										
	Serum/urine sample		O	O	O	O	O	O	O	O	O	O	O

^a^Kidney ultrasonography is performed annually for patients with CAKUT and every 3 years for other patients

^b^KEDI-WISC (5–15 years) or K-WAIS (16 years or older) [33]. ^c^Child Behavioral Checklist (CBCL) [34], ADHD Rating Scale-IV [35], State-Trait Anxiety Inventory (STAI) [36], and Children’s Depression Inventory (CDI) [37]. ^d^Parental bonding instrument [38]

Serum creatinine, blood urea nitrogen, cystatin C, parathyroid hormone, vitamin D, and urine chemistry (creatinine, urea, electrolyte, protein and microalbumin) are measured at a central laboratory (LabGenomics, SeongNam-si, South Korea) within one day after sampling to avoid inter-laboratory variation. At the central laboratory, serum creatinine is measured using the isotope dilution mass spectrometry (IDMS)-traceable method, and cystatin C is determined using immunonephelometry. Change in kidney volume is estimated by serial ultrasonography as previously reported [31]. In a subset of the participants, ambulatory blood pressure monitoring is performed in addition to the casual office blood pressure measurement. Isotopic GFR is measured in a subset of the participants to compare the validity of equations to estimate GFR in Korean children, including the bedside CKiD equation, complete CKiD equation, and Flanders Metadata equation [27, 32]. The patient’s quality of life is assessed using Pediatric Quality of Life (PedsQoL), and behavioral and neuropsychological statuses are evaluated using relevant methods at regular intervals (Table 3) [33–40].

A DNA sample is collected at the time of enrollment in the study. Serum and urine specimens are collected annually and stored in a deep freezer for further studies.

### Outcome variables

The primary endpoints of KNOW-Ped CKD are the progression of CKD, which is defined as a decline of eGFR by ≥50 %; a requirement for renal replacement therapy; and death. The secondary outcomes include the development of left ventricular hypertrophy (left ventricular mass/height^2.7 >95th percentile for age and sex), hypertension (>95th percentile for age, sex and height), impairment in growth (height Z score <−1.88 or weight Z score <−1.65), deviation from the Korean average ranges of neuropsychological and behavioral status, stunted growth of renal volume, and quality of life as well as changes from the baseline values.

### Statistical methods and analysis plan

The continuous variables of characteristics are presented using descriptive statistics with mean ± standard deviation for continuous variables and number and proportion for categorical variables. These variables will be compared using a one-way analysis of variance or Kruskal-Wallis test for the continuous variables and a chi-squared test or Fisher’s exact test for the categorical variables as appropriate. The time-to-event analysis will be the primary statistical method for analyzing the relationship between clinical characteristics and outcome variables, such as the progression of CKD, development of left ventricular hypertrophy, development of hypertension and impairment of growth. To analyze the primary outcome, we will apply the sub-distribution hazard models to our dataset with the competing risk event (progression of CKD or death without CKD) to evaluate the relative effects of covariates and cumulative incidence function [41].

### Interim report of baseline characteristics

From July 2011 to December 2013, 322 patients (218:104) were enrolled in the study. The average age at the time of enrollment was 10.0 ± 5.4 years (yrs). The etiologies of CKD included congenital renal dysplasia/hereditary disease (49 %), reflux nephropathy/chronic pyelonephritis (22 %), primary glomerular diseases (16 %), secondary glomerular diseases (4 %), and others. The stages of CKD were I for 15 %, II for 19 %, IIIa for 17 %, IIIb for 17 %, IV for 19 %, and V for 13 % of the cases. The following proportions of participants according to age group were noted: 10 % of patients were <2 years, 16 % were 2 to 5 years, 28 % were 6 to 11 year, and 47 % were 12 to 19 years. One-third of enrolled patients had histories of urinary tract infection, and 9 % exhibited developmental delay. At baseline, hypertension was noted in 27 % of patients, hyperlipidemia in 49 %, anemia in 45 %, and iron deficiency in 25 %. Growth delay was observed even in the early stage of CKD, with mean height and weight Z scores of −0.88 and −0.91, respectively, especially in younger (<6 years) patients with advanced CKD (IV and V). The baseline characteristics of the participants are summarized in Table 4.

**Table 4:** Clinical characteristics of KNOW-Ped CKD

CKD Stage	Total	I	II	IIIa	IIIb	IV	V
Patient Number	322	50 (15.5)	61 (18.9)	54 (16.8)	56 (17.4)	60 (18.6)	41 (12.7)
M:F (Male, %)	218:104 (67.7)	31:19 (62.0)	43:18 (70.5)	33:21 (61.1)	36:20 (64.3)	42:18 (70.0)	33:8 (80.5)
Age (years)	10.00 ± 5.45	7.04 ± 4.22	10.39 ± 5.61	11.50 ± 5.08	9.95 ± 5.36	9.48 ± 5.71	11.90 ± 5.42
Primary Kidney Disease							
CAKUT	198 (61.49)	17 (34.00)	38 (62.3)	30 (55.56)	41 (73.21)	43 (71.67)	29 (70.73)
GN	93 (28.88)	31 (62.00)	16 (26.23)	15 (27.78)	12 (21.43)	10 (16.67)	9 (21.95)
Others	31 (9.63)	2 (4.00)	7 (11.48)	9 (16.67)	3 (5.36)	7 (11.67)	3 (7.32)
Age Group							
<2 years	31 (9.63)	6 (12.00)	6 (9.83)	2 (3.70)	4 (7.14)	9 (15.00)	4 (9.76)
2–5 years	50 (15.53)	13 (26.00)	12 (19.67)	8 (14.81)	8 (14.29)	7 (11.67)	2 (4.88)
6–11 year	91 (28.26)	21 (42.00)	8 (13.11)	12 (22.22)	20 (35.71)	20 (33.33)	10 (24.39)
12–19 years	150 (46.58)	10 (20.00)	35 (57.373)	32 (59.26)	24 (42.856)	24 (40.00)	25 (60.98)
Growth, z score							
Height	−0.88 ± 1.43	−0.42 ± 1.09	−0.49 ± 1.41	−0.61 ± 1.32	−1.11 ± 1.22	−1.33 ± 1.62	−1.43 ± 1.58
Weight	−0.91 ± 1.65	−0.24 ± 1.39	−0.56 ± 1.69	−0.67 ± 1.34	−1.01 ± 1.4	−1.68 ± 1.88	−1.34 ± 1.7
eGFR (mL/min/1.73 m^2^)	53.1 ± 40.22	127.39 ± 36.16	68.98 ± 10.21	52.28 ± 4.57	36.03 ± 4.7	20.87 ± 5.42	10.46 ± 3.02
Hemoglobin (g/dL)	12.1 ± 1.95	12.49 ± 1.21	13 ± 2.1	12.61 ± 1.94	12.34 ± 1.89	11.19 ± 1.6	10.65 ± 1.74
Transferrin saturation (%)	27 ± 16	29 ± 28	26 ± 13	27 ± 13	28 ± 13	25 ± 12	29 ± 12
Cholesterol (mg/dL)	183 ± 59	197 ± 86	175 ± 46	190 ± 50	179 ± 574	184 ± 504	171 ± 61
Intact PTH (pg/mL)	119 ± 178	40 ± 39	46 ± 22	66 ± 47	99 ± 857	193 ± 217	302 ± 327
25(OH)D3 (ng/mL)	20.86 ± 10.09	20.02 ± 8.75	20 ± 10.62	21 ± 9	22 ± 9	21 ± 11	19 ± 10
u Protein/Cr (mg/mg)	1.77 ± 5.95	3.76 ± 14.36	0.8 ± 2.09	1.17 ± 2.08	1.44 ± 2.29	1.9 ± 2.01	1.88 ± 1.52
LVMI (g/m^2.7^)	70 ± 22	71 ± 21	71 ± 23	65 ± 18	66 ± 17	70 ± 24	80 ± 26

## Discussion

KNOW-Ped CKD is a prospective cohort study on Asian pediatric CKD with long-term follow-up. Although several investigations of school urinary screening and CKD have been reported, comprehensive studies on CKD have been rare in Asian countries [24, 42–49]. To the best of our knowledge, only one prospective study has been performed, involving 447 Japanese children with CKD stages III-V with a relatively short follow-up period [43]. Therefore, primary renal disease and progression rates in the earlier stages of CKD and their risk factors for progression over an extended period have yet to be investigated in Asian children. The CKiD study, a leading, comprehensive, prospective cohort study of pediatric CKD, indicated that non-Caucasian race was a determinant of proteinuria, which was subsequently a prognostic factor for CKD progression, implying a difference in progression among races for pediatric CKD [22, 50]. Additionally, outstanding pediatric CKD cohort studies, namely the CKiD study and the 4C study, as well as the Japanese national study, enrolled children with decreased eGFR, which excluded those with CKD stages I and II. In KNOW-Ped CKD, Korean children with CKD of stages I to V are enrolled.

Although advanced CKD tends to progress to ESRD, many children remain at stage I or II for a long time. The risk factors for progression in these patients remain unknown. In some children, at least transiently, renal function appears to recover. With the KNOW-Ped CKD study, we will investigate the determinants of prognosis for CKD stages I and II. Additionally, annual measurements of the kidney size in this study will answer the following questions: 1) Does the kidney volume in CKD correlate with renal function? 2) How much kidney growth is necessary for functional improvement? With KNOW-Ped CKD, we will be able to delineate the characteristics of Asian pediatric CKD, which will lead to a better understanding of the entire progression of CKD in children. We will also identify the similarity and differences among different ethnicities in pediatric CKD, enabling us to customize therapeutic management.

Another characteristic of KNOW-Ped CKD is that this study enrolls the majority of pediatric pre-dialysis CKD patients in Korea. According to the data from Health Insurance Review and Assessment Service of the Korean National Health Insurance Program, a unique and obligatory public healthcare program in Korea, CKD patients <20 years of age numbered approximately 700 in 2014 [51]. Excluding the number of ESRD under renal replacement therapy, KNOW-Ped CKD is expected to enroll more than half of the total Korean pediatric CKD patients with the participation of seven major pediatric nephrology centers. Therefore, this cohort study will reflect the current status of pediatric CKD in Korea fairly accurately.

Staging of CKD in KNOW-Ped CKD is based on the bedside CKiD equation. Although this equation has not yet been validated in Korean children, the bedside CKiD equation is simple and easy and therefore useful in clinical practice. Therefore, the authors expect that findings based on this equation, after validation via the KNOW-Ped CKD study, can be readily applied in patient care. Measuring GFR using the clearance of injected isotopic material is performed in a subset of the participants to investigate whether known pediatric eGFR estimation equations are applicable in Korean children. Regarding the staging of CKD, categorizing CKD stages in older children cannot be applied for children <2 years because the normal GFR is lower in children <2 years of age [26]. Although the KDIGO guidelines suggest classifying normal, moderate, or severe reduction in GFR for children <2 years old, staging of CKD reflecting kidney growth is necessary to assess the progression of CKD. Therefore, we defined operational GFR cut-offs as follows: stage I: eGFR ≥75 % of normal GFR for age; stage II: eGFR 60 to 74 % of normal GFR for age; stage IIIa: eGFR 45 to 59 % of normal GFR for age; stage IIIb: eGFR 30 to 44 % of normal GFR for age; stage IV: eGFR 15 to 29 % of normal GFR for age; and stage V: eGFR <15 % of normal GFR for age [29, 52, 53]. This operational definition should be evaluated and possibly modified throughout this study given that it has not been validated. Additionally, when interpreting the results, it must be considered that not all of the formulas for eGFR estimation are generated by creatinine values measured using the same method.

Three years after launching the KNOW-Ped CKD study, more than 300 children with CKD have participated in this study. Additional analysis will be conducted upon the completion of enrollment; however, the distribution of the etiology of CKD is similar to that of previous reports, including CKiD and the Japanese nationwide survey study [24, 54]. The CKD complications of hypertension, growth retardation, anemia, and metabolic derangement were observed as expected. These findings reveal that our current management of pediatric CKD in Korea has room for improvement, such as early evaluation and intervention for iron deficiency in this population.

Although this study has some limitations, such as the use of the operational definition of CKD staging for younger patients and eGFR equations that have not yet been verified in Korean children, we expect that KNOW-Ped CKD, a prospective cohort study in pediatric CKD for a long-term period in an Asian country, will reveal more comprehensive characteristics of pediatric CKD, especially in its earlier stage, and the risk factors of progression that can be translated into better management of these patients through international collaboration with existing cohort studies.

## Ethics approval and consent to participate

This study was approved by the institutional review boards of the participating centers, namely Seoul National University Hospital, Asan Medical Center, Kyungpook National University Hospital, Severance Hospital, Samsung Medical Center, Jeju University Hospital, and Pusan National University. Children <20 years of age with CKD are enrolled in this study after informed consent is obtained from parents or guardians.

## Consent for publication

Not applicable.

## Availability of data and material

All data is included within the article.

## Abbreviations

CAKUT: congenital anomalies of the kidney and urinary tract
CKD: chronic kidney disease
eGFR: estimated glomerular filtration rate
ESRD: end-stage renal disease
GFR: glomerular filtration rate
IDMS: isotope dilution mass spectrometry
KDIGO: Kidney Disease Improving Global Outcomes
KNOW-Ped: CKD KoreaN cohort study for Outcomes in patients With Pediatric CKD
NAPRTCS: North American Pediatric Renal Trials and Collaborative Studies
PedsQoL: quality of life assessed with Pediatric Quality of Life
RRT: renal replacement therapy
